# Adverse Employment Histories and Later Health Functioning: European Findings Based on Life Histories From SHARE and ELSA

**DOI:** 10.1093/geroni/igaa057.1844

**Published:** 2020-12-16

**Authors:** Christian Deindl, Morten Wahrendorf

**Affiliations:** 1 Heinrich-Heine-Universität Düsseldorf, Duesseldorf, Nordrhein-Westfalen, Germany; 2 Centre for Health and Society, Institute of Medical Sociology, Medical Faculty, University of Düsseldorf, Duesseldorf, Nordrhein-Westfalen, Germany

## Abstract

We investigate associations between adverse employment histories over time and health functioning in later life, and explore moderation by national labor market policies. Harmonized life history data come from two studies, SHARE and ELSA, with health beyond age 50 (men= 11,621; women= 10,999). Adverse employment histories consist of precarious, discontinued and disadvantaged careers between age 25 and 50, and we use depressive symptoms, grip strength and verbal memory as outcomes. Adverse employment histories are associated with poor health functioning later in life, especially repeated periods of unemployment, involuntary job losses, weak labor market ties and disadvantaged occupational positions. We find no variations of the associations by national labor market policies. Our study highlights the need to improve working conditions at early career stages. Despite the importance in shaping employment histories, the role of national policies in modifying the impact of employment on health is less clear.

